# Effects of chronic static stretching interventions on jumping and sprinting performance–a systematic review with multilevel meta-analysis

**DOI:** 10.3389/fphys.2024.1372689

**Published:** 2024-03-26

**Authors:** Konstantin Warneke, Patrik Freundorfer, Gerit Plöschberger, David G. Behm, Andreas Konrad, Tobias Schmidt

**Affiliations:** ^1^ Institute of Human Movement Science, Sport and Health, University of Graz, Graz, Austria; ^2^ Institute of Sport Science, University of Klagenfurt, Klagenfurt am Wörthersee, Austria; ^3^ Institute of Interdisciplinary Exercise Science and Sports Medicine, MSH Medical School Hamburg, Hamburg, Germany; ^4^ School of Human Kinetics and Recreation, Memorial University of Newfoundland, St. John’s, NL, Canada

**Keywords:** long-term, athletic performance, stretch-shortening cycle, plyometrics, speed strength, stretch training, static stretching

## Abstract

When improving athletic performance in sports with high-speed strength demands such as soccer, basketball, or track and field, the most common training method might be resistance training and plyometrics. Since a link between strength capacity and speed strength exists and recently published literature suggested chronic stretching routines may enhance maximum strength and hypertrophy, this review was performed to explore potential benefits on athletic performance. Based on current literature, a beneficial effect of static stretching on jumping and sprinting performance was hypothesized. A systematic literature search was conducted using PubMed, Web of Science and Google scholar. In general, 14 studies revealed 29 effect sizes (ES) (20 for jumping, nine for sprinting). Subgroup analyses for jump performance were conducted for short- long- and no stretch shortening cycle trials. Qualitative evaluation was supplemented by performing a multilevel meta-analysis via R (Package: metafor). Significant positive results were documented in six out of 20 jump tests and in six out of nine sprint tests, while two studies reported negative adaptations. Quantitative data analyses indicated a positive but trivial magnitude of change on jumping performance (ES:0.16, *p* = 0.04), while all subgroup analyses did not support a positive effect (*p* = 0.09–0.44). No significant influence of static stretching on sprint performance was obtained (*p* = 0.08). Stretching does not seem to induce a sufficient stimulus to meaningfully enhance jumping and sprinting performance, which could possibly attributed to small weekly training volumes or lack of intensity.

## 1 Introduction

Due to its paramount importance in team sports as well as track and field, a vast amount of research was performed to ascertain training routines to improve jumping and sprinting performance (Ramirez-Campillo et al., 2020; Byrne et al., 2022; Patti et al., 2022; Ramirez-Campillo et al., 2022). Indeed, authors described speed strength to be game-changing in soccer (Requena et al., 2014; Keiner et al., 2022b), basketball (Brughelli et al., 2008; Delextrat and Cohen, 2008; Delextrat and Cohen, 2009), or handball (Manchado et al., 2013; Wagner et al., 2014), underlining the relevance of developing appropriate and effective training routines. In accordance with the literature, several training routines included plyometric exercises (Markovic and Newton, 2007; Morris et al., 2022), while other studies suggested maximal strength capacity to positively influence speed strength (Lesinski et al., 2016; Wirth et al., 2016).

Accordingly, a high influence of lower body maximal strength in the squat was frequently reported. With r = 0.78, Wisloff et al. (Wisløff et al., 2004) reported a significant relationship between maximum strength in half squats and vertical jump performance in soccer players (Wisløff et al., 2004) while Warneke et al. (2022c) showed maximum strength in the deep squat and deadlift to be strongly correlated with vertical and horizontal jumping performance as well as linear and change of direction sprints. Therefore, it does not seem surprising that chronic resistance training routines were sufficient to meaningfully enhance speed strength performance in athletic populations (Behm et al., 2017; Lohmann et al., 2022).

Even though resistance training can be considered safe and effective, it is necessary to supervise the training to avoid poor exercise technique and inappropriate training loading (Kraemer and Ratamess, 2004; Faigenbaum and Myer, 2010). Therefore, developing facility and time independent, safe, and effective training alternatives seem to be beneficial (Morie et al., 2010; Schwendinger and Pocecco, 2020). Interestingly, recently published articles suggest high intensity (Panidi et al., 2023) or high volume (Arntz et al., 2023) static stretching can induce muscle hypertrophy and maximal strength increases, that were similar to resistance training adaptations, if performed appropriately (Warneke et al., 2023b). These results are in accordance with earlier research, indicating potential performance increases in response to chronic stretching routines (Shrier, 2004; Medeiros and Lima, 2017). Since Behm et al. (2023) also suggested static stretching to be a possible alternative to heavy strength training programs, the question arises about the practical relevance when aiming to enhance athletic performance. No previous systematic reviews have explored the possibility to induce meaningful improvements in jumping height and sprinting speed. As a consequence, this systematic review with meta-analysis was conducted to investigate the possibility of using static stretching to enhance the listed parameters in healthy active participants. Based on the current literature showing stretch-induced strength and muscle size increases, it was hypothesized that static stretching could provide a sufficient stimulus to enhance jumping and sprinting performance.

## 2 Materials and methods

Adhering to the PRISMA Guidelines (Page et al., 2021) (see Supplemental Material) a systematic literature search was conducted on the PubMed, Web of Science and Scopus databases, which was supplemented by the first 500 Google Scholar hits. The study did not match the study registration criteria of the PROSPERO database (i.e., health-related outcomes) as it investigated athletic performance outcomes. Therefore, the study protocol was not registered.

### 2.1 Eligibility criteria

After removing duplicates remaining study titles and abstract were screened using the following eligibility criteria. Studies were excluded if they 1) did not include a passive control group or 2) incorporated older participants (>65 years), patients or injured participants 3) investigated acute effects of stretching 4) did not perform static stretching. The cut-off to discriminate younger from old adults was set under consideration of the common World Health Organization (WHO) classification. Accordingly, adhering to the PICO (participant/patient, intervention, comparison, outcome) guidelines (Eriksen and Frandsen, 2018), the search term was built considering the following inclusion criteria:

**P**articipants: young and healthy participants.

**I**ntervention: at least 2 weeks of static stretching interventions.

**C**omparison/control: controlled study design using a passive control (without receiving an intervention instruction within the study).

**O**utcome: any type of jumping or sprinting performance, including change of direction and linear sprint testing.

#### 2.1 1 Literature search

The literature search was performed in March 2023 and updated in November 2024. Three authors (PF, GP and TS) independently reviewed the search results. The search was conducted using the following search term.

### 2.2 Study selection

After performing the independent systematic literature search, each study selected for inclusion was additionally reviewed considering the inclusion criteria by another author (KW), who extracted the data afterwards. The final excel sheets were independently double checked by the authors (GP, PF).

### 2.3 Risk of bias

Quality assessment was performed following the “PEDro scale” criteria (Maher et al., 2003; de Morton, 2009). The quality assessment was independently performed by two authors (PF, TS), and in the case of differing results, a third reviewer decided (KW). The risk of publication bias was assessed by visually inspecting funnel plots.

GRADE working group criteria (Atkins et al., 2004) were applied to rate the certainty of evidence following categorizations of “very low” (effect estimate very uncertain), “low” (further research very likely changing the effect estimate), “moderate” (further research likely to change the effect estimate) or “high” (further research very unlikely to change the effect estimate). In accordance to the described criteria and the inclusion of (randomized) controlled trials, the quality of evidence was initially classified as high and adjusted afterwards based on possible study limitations (risk of bias). Attributes like inconsistency, uncertainty of directness, or imprecise data, reporting bias caused a downgrading, while strong evidence of association, evidence of a dose-response gradient and plausible confounders upgraded the certainty of evidence.

### 2.4 Statistical procedure

Data were extracted by PF and KW and double checked by GP. If no original values (means (M) and standard deviations (SD)) were provided in the full text, the corresponding authors were contacted. If no answer was received, values were imputed from graphics, if applicable. Otherwise, the study was not further considered for further calculation. Using collected M and SD from pre- and post-tests, changes were calculated, performing M_(posttest)_–M_(pretest)_. Standard deviations were pooled as ${SD}_{pooled}=\sqrt{\frac{\left(n_{1}-1\right)*{SD}_{1}^{2}+\left(n_{2}-1\right)*{SD}_{2}^{2}}{\left(n_{1}-1\right)+\left(n_{2}-1\right)}}$ .

Accounting for dependency of effect size with multiple outcomes, a multilevel meta-analysis was performed to pool the standardized mean differences (SMD) and 95% confidence intervals (CI) plotting the stretching intervention *versus* the passive control condition. Furthermore, subgroup analyses were performed for jump performance, distinguishing between the short stretch shortening cycle (SSC) activities by including a variety of drop jumps, the long SSC with different countermovement jumps and jumps without any assumed SSC like the squat jump. Outcome heterogeneity is expressed using τ^2^ for inter- and intragroup heterogeneity and is plotted in the graphical ES illustration. Pooled effect sizes (ES) were interpreted as follows: 0≤ES < 0.2 trivial, 0.2≤ES < 0.5 small, 0.5≤ES < 0.8 moderate and ES ≥ 0.8 large (Faraone, 2008). All calculations were performed with R using the package metafor to account for outcome dependencies of multiple outcomes.

## 3 Results

**FIGURE 1:**
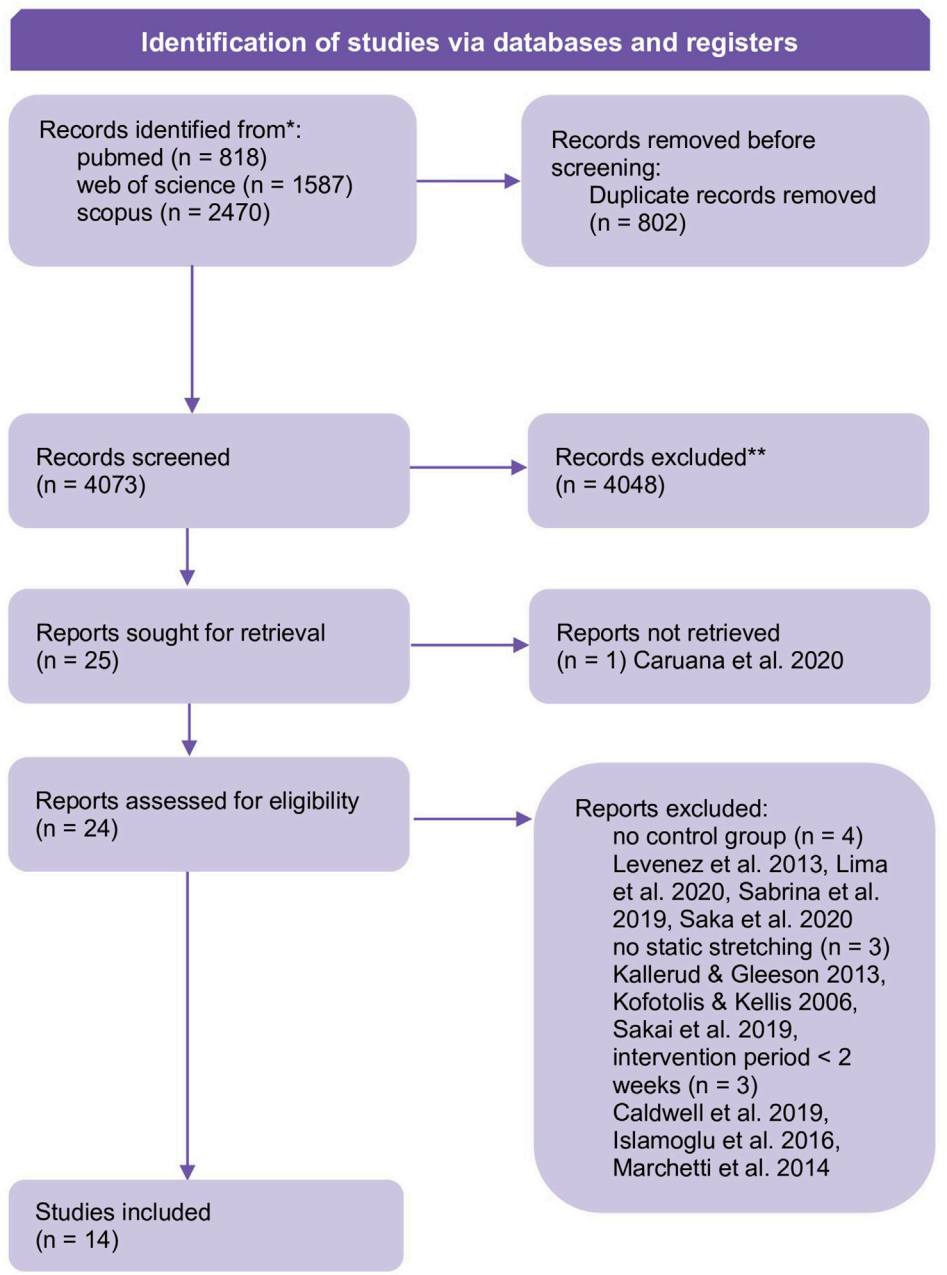
PRISMA flow chart adhering to Page et al. (2021) to illustrate the systematic literature search.

The literature search resulted in 14 articles (Figure 1). The quality assessment is illustrated in Table 1, study characteristics are provided in Table 2.

**TABLE 1:** Study characteristics of the included research.

Study	Parameter	Participants	Stretch intervention	Testing condition
Alipasali et al. (2022)	CMBJ	n = 42	6 weeks	Two JVC 9800 video cameras for height assessment, Ariel Performance Analyses System, 19 body landmarks for 3D software
		sport students	stretching of lower limbs	
		3 groups: n = 14	3 times per week	
		SS, DS, CG	2 sets with 6 exercises and	
			10 s stretch	
			Intensity	
			maximum joint ROM without pain	
Donti et al. (2021)	Single-leg CMJ	n = 30	9 weeks	Knee bent to approximately 90°
		Child female gymnasts	Quadriceps stretch	Hands akimbo throughout jump
		2 groups	3 times per week	Extended body position at takeoff and landing
		SG: n = 19	One leg 3 × 30 s stretch and 30 s rest, other leg 90 s single static stretch	Jumping height was
		CG: n = 11	Intensity	determined from flight-time
		One leg was intermittently and the other continuously stretched	8 on 0–10 Wong-Baker FACES Pain Scale	using Optojump system
Hunter and Marshall (2002)	CMJ, DJ 30, DJ 60, DJ 90	n = 50	10 weeks (40 sessions)	CMJ, DJ30, DJ60, DJ90 hands on hips no restrictions placed on countermovement or ground contact time calculation via force plate, Bertec 6090, Bertec Corporation
		men with variety of sporting backgrounds	of lower-body stretching	
		4 groups	4 days per week	
		Group *p*: n = 11	duration of one stretch increases from	
		Group S: n = 11	week 1 + 2: 20 s	
		Group PS: n = 14	week 3 + 4: 30 s	
		Group C: n = 14	week 5 + 6: 40 s	
			week 7 + 8: 50 s	
			week 9 + 10: 60 s	
			each stretch 3 repetitions	
			12 exercises	
			PS Group	
			same stretching protocol + two power training sessions per week	
			Intensity	
			stretches to mild discomfort	
Ikeda & Ryushi, (2021)	SJ CMJ RJ	n = 25 men	6 weeks knee extensors	CMJ and SJ: Best of 3 jumps (each) RJ: 6 consecutive jumps as high as possible with shortest possible ground contact time, first jump does not count, highest value out of the 5 jumps taken for RJ calculations calculated via Kistler force plate, Type 9285 A
		no athletes but sports background	Stretch	
		2 groups	3 times per week	
		SG, CG	6 sets of 30s stretch and 60s rest between sets	
			Intensity	
			maximum stretch for knee extensors but within the limit of pain	
Nakamura et al. (2021)	Single-leg DJ (20-cm)	n = 40 men	4 weeks (12 sessions)	20-cm box single-leg drop jump on Jump mat system, 4Assist, hands crossed in front of the chest
		3 groups	3 times per week	
		HI-SS: n = 14	3 sets of 60 s stretch and 30 s rest using a stretching board	
		LI-SS:n = 13	Intensity	
		CON: n = 13	11-point numerical scale was used (0 = none to 11 = very painful)	
			HI-SS at 6–7	
			LI-SS at 0–1	
Panidi et al. (2021)	Single leg CMJ	n = 21 female volleyball player single leg intervention, the other leg used for control	12 weeks (60 sessions)	one-leg CMJ Force platform Applied Measurements Ltd. WP800 arms akimbo and vertical jump with ankle and knee fully extended
			static stretching of plantar flexors	
			5 times per week	
			2 sets with 6 exercises	
			week 1–3: 45 s	
			week 4–6: 60 s	
			week 6–12: 75 s	
			Intensity 8–9 on	
			Wong Baker FACES Pain Scale	
			near point of discomfort	
Warneke et al. (2022d)	CMJ DJ (24-cm) SJ	n = 35	6 weeks	jumps performed on
		healthy students from physical education programs	(daily stretch for 10 min Per leg/42 sessions)	Force plate AMTI, BP600900-4000
		2 groups	stretching of the plantar flexors with Stretching Board	SJ initiated from squat position
		IG: n = 17	Intensity	after 2s hold without momentum
		rPA: n = 18	stretching pain of 7–8 out of 10 on a numeric pain scale	CMJ and SJ, hands on hips DJ (from 24 cm height) best of five trials with hands on hips
Yuktasir and Kaya (2009)	Drop jump (60 cm)	n = 28	6 weeks (24 sessions)	DJ from 60 cm flight time measured with Newtest 1000
		men from sports school	Hamstring stretching	
		3 groups	4 times per week	
		SS: n = 10	30 s stretch, 4 sets	
		PNF: n = 9	Intensity	
		CG: n = 9	Not exceeding 5 on	
			Standard visual analogue scale (VAS)	
Barbosa et al. (2020)	jump performance: triple hop sprint performance: 20-m sprint	n = 45	3 weeks (until 10 sessions completed)	triple hop
		healthy and active men	stretching of hamstring muscles	single non dominant leg hop
		3 groups	3 times per week, 3 sets of 30 s stretch and 30 s rest	with arm swinging, measured from starting line to heel landing mark
		SS: n = 15	Intensity: until discomfort sensation on the hamstrings	three trials with 30-s recovery
		DS: n = 15		2 min rest between triple hop an sprint
		CG: n = 15		20-m sprint: participants start 0.5 m behind with non-
Bazett-Jones et al. (2008)	jump performance	n = 21	6 weeks (24 sessions)	Vertical Jump
		female track and field athletes (3rd division)	of hamstring stretching	hands on hips - no arm-swing movement two jumps and average of both used for analysis Kistler Quattro Jump Force Plate
		2 groups	4 consecutive days per week	55-m sprint
		SG: n = 10	4 sets of 45 s stretch each leg	three- or four-point-position with one finger on the timing device Speedtrap II Wireless Timing System
		CG: = 11	Intensity: until mild discomfort in hamstring muscle group	single test
Hadjicharalambous (2016)	jump performance via broad jump sprint performance: 10-m 35-m sprint Illinois Agility test	n = 23	4 weeks (32 sessions)	Broad Jump: with arm swing and countermovement from standing position examined with 10-m/35-m sprint/Agility test: evaluated with Microgate, Racetime2 kit-system and photoelectric cells set at chest height all tests performed on grass pitch
		healthy young football players on professional niveau	stretching of lower body	
		FG: n = 11	2 times per day, 4 days per week	
		CG:n = 12	stretching durations	
			week 1: 15 s	
			week 2: 20 s	
			week 3 + 4: 30 s	
			Intensity	
			point of mild discomfort but no pain	
Kokkonen et al. (2007)	jump performance: vertical jump, standing long jump sprint performance: 20-m sprint	n = 38 students	10 weeks (30 sessions)	vertical jump
		physically inactive or recreationally active	static stretching of the major lower-extremity muscle groups	Vertec testing device (Questtek Corp.)
		2 groups	3 days a week	standing long jump
		STR: n = 19	15 different exercises	5 jumps submaximal for warm-up then best of three with 1 min rest between jumps
		CON: n = 19	3 sets of 15 s stretch	20-m sprint
			Intensity	5 min rest after long jump before sprint
			stretching without pain	standing start and measured with Speedtrap II, Brower Timing Systems 5 submaximal warm-up sprints allowed best of three with 3 min rest between sprints
Alipasali et al. (2019)	sprint performance: 4.5-m sprint, 9-m sprint	n = 27 men	6 weeks (18 sessions)	4.5-m and 9-m sprint: one leg 40 cm behind starting line Best of two used with 3 min rest between each sprint, sprints measured by dual-beam photocell system, Autonics Beam Sensor BL5M-MFR and digital timer, Saint Wien Digital Timer Ty H5K
		volleyball player regional level	stretching of lower limbs	
		3 groups	3 times per week	
		SG: n = 11	2 sets with 10 s stretch	
		DG: n = 7	Intensity	
		CG: n = 9	maximum joint ROM without pain	
Rodriguez Fernandez et al. (2016)	sprint performance: 30-m sprint	n = 103	7 weeks (42 sessions)	Linear 30-m sprint outdoor assessed with Photoelectric ell DSD Laser System, DSD Inc. Foot positioned 50 cm behind start line
		from 5 teams u18 top regional category	static stretching of hamstring muscles	
		2 groups	6 days a week	
		CG: n = 22	4 exercises at the end of training 2 × 30 s stretch	
		E.G.,: n = 81	Intensity	
			stretching exercises without pain	

DJ, drop jump (height), SJ, squat jump; RJ, rebound jump CMBJ, counter-block-movement jump, CMJ, countermovement jump; VJ, vertical jump; SLJ, standing long jump; BJ, broad jump; SS, static stretching; DS, dynamic stretching; CG, control group, Group *p* = power training group, Group S = flexibility training group, Group PS, flexibility and power training group, Group C = control group, HI-SS, high intensity static stretch group; LI-SS, low intensity static stretch group; CON, Non-intervention control group; IG, intervention group; rPA, reduced physical activity group; PNF, proprioceptive neuromuscular facilitation group; FG, flexibility group; STR, stretching group (intervention group), E.G.,, experimental group (intervention).

**TABLE 2:** Provides the results of the quality assessment using the PEDro Scale.

Parameter	Author and publication year	1. Specific eligibility criteria	2. Randomly allocated to groups	3. Allocation was concealed	4. Groups similar baseline	5. Blinding of subjects	6. Blinding of therapists	7. Blinding of assessors	8. Measured one key outcome from >85%	9. Received treatment or control condition as allocated or analysed by “intention to treat”	10. Statistical comparisons of between-group results	11. Point measures and measures of variability	Results (first question not included)
jump	Alipasali et al. (2022)	Y	Y	N	Y	N	N	N	Y	Y	Y	Y	6 of 10
	Donti et al. (2021)	Y	Y	N	Y	N	N	N	Y	Y	Y	Y	6 of 10
	Hunter and Marshall (2002)	Y	Y	N	Y	N	N	N	N	N	Y	Y	4 of 10
	Ikeda & Ryushi, (2021)	Y	Y	N	Y	N	N	N	Y	Y	Y	Y	6 of 10
	Nakamura et al. (2021)	Y	Y	N	Y	N	N	N	Y	Y	Y	Y	6 of 10
	Panidi et al. (2021)	Y	Y	N	Y	N	N	Y	N	Y	Y	Y	6 of 10
	Warneke et al. (2022d)	Y	N	N	Y	N	N	N	Y	Y	Y	Y	5 of 10
	Yuktasir & Kaya, (2009)	Y	Y	N	Y	N	N	Y	Y	Y	Y	Y	7 of 10
jump and sprint	Barbosa et al. (2020)	Y	Y	Y	Y	N	N	Y	Y	Y	Y	Y	8 of 10
	Bazett-Jones et al. (2008)	Y	Y	N	Y	N	N	Y	Y	Y	Y	Y	7 of 10
	Hadjicharalambous (2016)	Y	Y	N	Y	N	N	N	Y	Y	Y	Y	6 of 10
	Kokkonen et al. (2007)	Y	Y	N	Y	N	N	Y	Y	Y	Y	Y	6 of 10
sprint	Alipasali et al. (2019)	Y	Y	N	Y	N	N	N	N	Y	Y	Y	5 of 10
	Rodriguez Fernandez et al. (2016)	Y	Y	N	Y	N	N	N	Y	Y	Y	Y	6 of 10

After excluding about 4000 articles based on title and abstract, the remaining 27 full texts were checked for eligibility. From the available 26 studies, four articles did not include a non-intervened passive control, while six others did not fulfill the minimum requirements of intervention periods or did not exclusively used static stretching (see Figure 1).

Eight studies exclusively examined jumping performance (Hunter and Marshall, 2002; Yuktasir and Kaya, 2009; Donti et al., 2021; Ikeda and Ryushi, 2021; Nakamura et al., 2021; Panidi et al., 2021; Alipasali et al., 2022; Warneke et al., 2022d), two studies (Rodriguez Fernandez et al., 2016; Alipasali et al., 2019) only sprint performance. Since four studies assessed both jumping and sprinting performance (Kokkonen et al., 2007; Bazett-Jones et al., 2008; Hadjicharalambous, 2016; Barbosa et al., 2020), the results regarding jumping performance from 12 studies and sprinting results from six studies were examined.

### 3.1 Qualitative study results

#### 3.1.1 Jumping performance

The intervention period ranged between three (Barbosa et al., 2020) and 12 weeks (Panidi et al., 2021) in which stretching sessions were scheduled at least 3 days/week (Kokkonen et al., 2007; Barbosa et al., 2020; Ikeda and Ryushi, 2021; Nakamura et al., 2021; Panidi et al., 2021) to a maximum of 7 days/week (Warneke et al., 2022d). In one study, stretching was also performed twice a day (Hadjicharalambous, 2016).

Stretching duration per session ranged from 10 s (Alipasali et al., 2022) to 10 min (Warneke et al., 2022d) and included a minimum of one (Warneke et al., 2022d) and a maximum of four (Bazett-Jones et al., 2008) sets. The stretching intensity was determined via subjective pain scales and ranged from stretching without pain (Alipasali et al., 2022) to stretching at the point of discomfort (Panidi et al., 2021) or the limit of pain (Ikeda and Ryushi, 2021).

Six studies included drop jumps from five different heights, six countermovement jumps (single leg in one study (Panidi et al., 2021), vertical jump and standing jump in two cases each and rebound jump, triple hop, standing long or broad jump in one result each. For quantitative analysis, the jumping types were distinguished via the expected stretch-shortening cycle length (short and long SSC subgroup).

For jumping performance, effect sizes showed jumping performance increases ranging from 2.3% (Kokkonen et al., 2007) to 27 ± 30% (Panidi et al., 2021). Twelve (12) trivial and/or non-significant effect sizes from - 3.84% (Bazett-Jones et al., 2008) to 16.0% (Donti et al., 2021) were observed, while two effect sizes showed performance decreases from the pre-to the post-test with −3.7% (Barbosa et al., 2020) to −7.93% (Ikeda and Ryushi, 2021).

#### 3.1.2 Sprint performance

Six studies were found investigating the effects of chronic stretching programs on sprint performance. A total of 257 subjects were included performing nine different sprint variants between 4.5 m (Alipasali et al., 2019) and 55 m (Bazett-Jones et al., 2008). Intervention durations ranged between 3 weeks (Barbosa et al., 2020) to 10 weeks (Kokkonen et al., 2007) stretching up to 6 days per week (Rodriguez Fernandez et al., 2016) with Hadjicharalambous (2016) stretching twice a day. Stretching durations varied between 10 s (Alipasali et al., 2019) and 45 s (Bazett-Jones et al., 2008). Exercises were performed repeatedly in two (Rodriguez Fernandez et al., 2016; Alipasali et al., 2019) to four (Bazett-Jones et al., 2008) sets, with rests ranging in duration from 10 s (Alipasali et al., 2019) up to 1 minute (Kokkonen et al., 2007; Bazett-Jones et al., 2008). The intensity of stretching was again determined by using individual pain perception, mostly reporting mild discomfort.

Six out of nine sprinting tests showed performance improvements of 1.3% (Kokkonen et al., 2007)–10% (Hadjicharalambous, 2016), while three tests did not reach the level of significance with 0.04% (Barbosa et al., 2020) to 2.2% (Hadjicharalambous, 2016) pre-to post-test changes.

### 3.2 Summary of the results

An overview for the results is illustrated in Table 3, providing an illustrated overview of the results considering the collected parameters and the described effects.

**TABLE 3:** Overview of direction of the effects, bright grey illustrates no significant change from pre-to post-test, no color (white) shows a significant improvement of the motor task while fields marked in dark grey showed stretching negatively impacted jumping or sprinting performance.

Parameter	Author and publication year	Static stretching effect									
Jump performance	Alipasali et al. (2022)	CMBJ									
	Donti et al. (2021)	CMJ									
	Hunter and Marshall (2002)	CMJ	DJ30*			DJ60*				DJ90*	
	Ikeda and Ryushi (2021)	CMJ			SJ				RJ		
	Nakamura et al. (2021)	DJ20									
	Panidi et al. (2021)	Single leg CMJ									
	Warneke et al. (2022d)	CMJ			SJ				DJ24		
	Yuktasir and Kaya (2009)	DJ60									
Jump and sprint performance	Barbosa et al. (2020)	Triple hop						20-m sprint			
	Bazett-Jones et al. (2008)	VJ						55-m sprint			
	Hadjicharalambous (2016)	BJ		10-m sprint			35-m sprint				Illinois agility test
	Kokkonen et al. (2007)	VJ			SLJ				20-m sprint		
Sprint performance	Alipasali et al. (2019)	4.5-m sprint						9-m sprint			
	Rodriguez Fernandez et al. (2016)	30-m sprint									

DJ, drop jump (height), SJ, squat jump; RJ, rebound jump CMBJ, counter-block-movement jump, CMJ, countermovement jump; VJ, vertical jump; SLJ, standing long jump; BJ, broad jump; SS, static stretching; DS, dynamic stretching; CG, control group, Group *p* = power training group, Group S = flexibility training group, Group PS, flexibility and power training group, Group C = control group, HI-SS, high intensity static stretch group; LI-SS, low intensity static stretch group; CON, Non-intervention control group; IG, intervention group; rPA, reduced physical activity group; PNF, proprioceptive neuromuscular facilitation group; FG, flexibility group; STR, stretching group (intervention group), E.G., experimental group (intervention).

### 3.3 Quantitative analysis of the extracted effects

**FIGURE 2:**
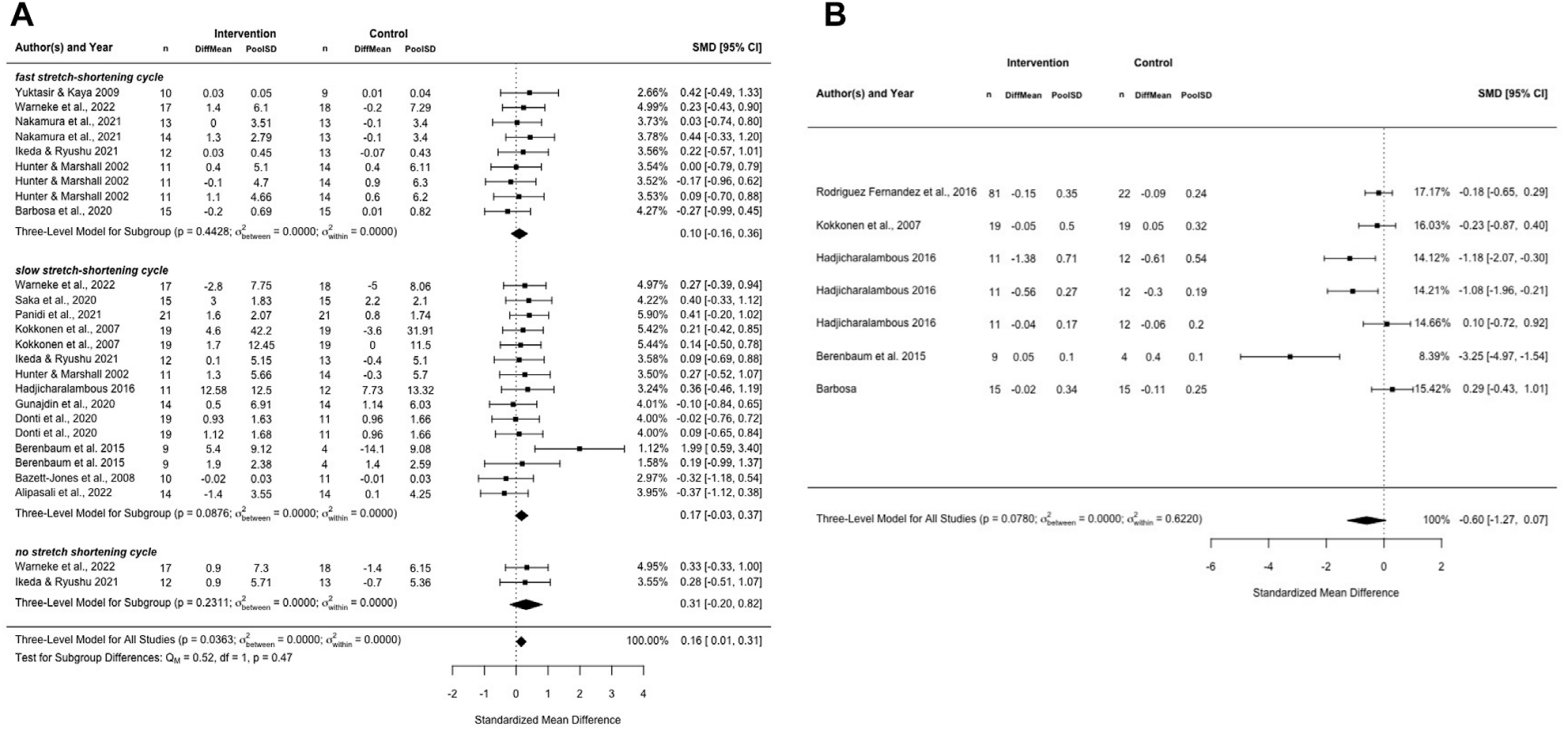
Forest plots for **(A)** chronic stretching on jumping performance, divided into fast stretch-shortening cycle, slow stretch-shortening cycle and no stretch-shortening cycle jumping actions and **(B)** chronic stretching on sprinting performance.

Quantitative analysis of the effects is illustrated in Figure 2, showing the forest plot for jumping performance (no, short, and long stretch-shortening cycle) (a) and sprinting performance (b).

Including 25 effects with *p* = 0.036 there was an overall trivial magnitude effect with ES = 0.16 (0.01–0.31) with a high certainty of evidence. The individual subgroup analyses failed to reach significance for jump performance using the fast SSC (ES = 0.10, *p* = 0.449), slow SSC (ES = 0.17, *p* = 0.09) and no SSC (ES = 0.31, *p* = 0.23). Due to limited number of extracted effect sizes, no further subgroup analysis was performed. With ES = −0.60, *p* = 0.078, no significant sprinting improvements can be assumed.

**FIGURE 3:**
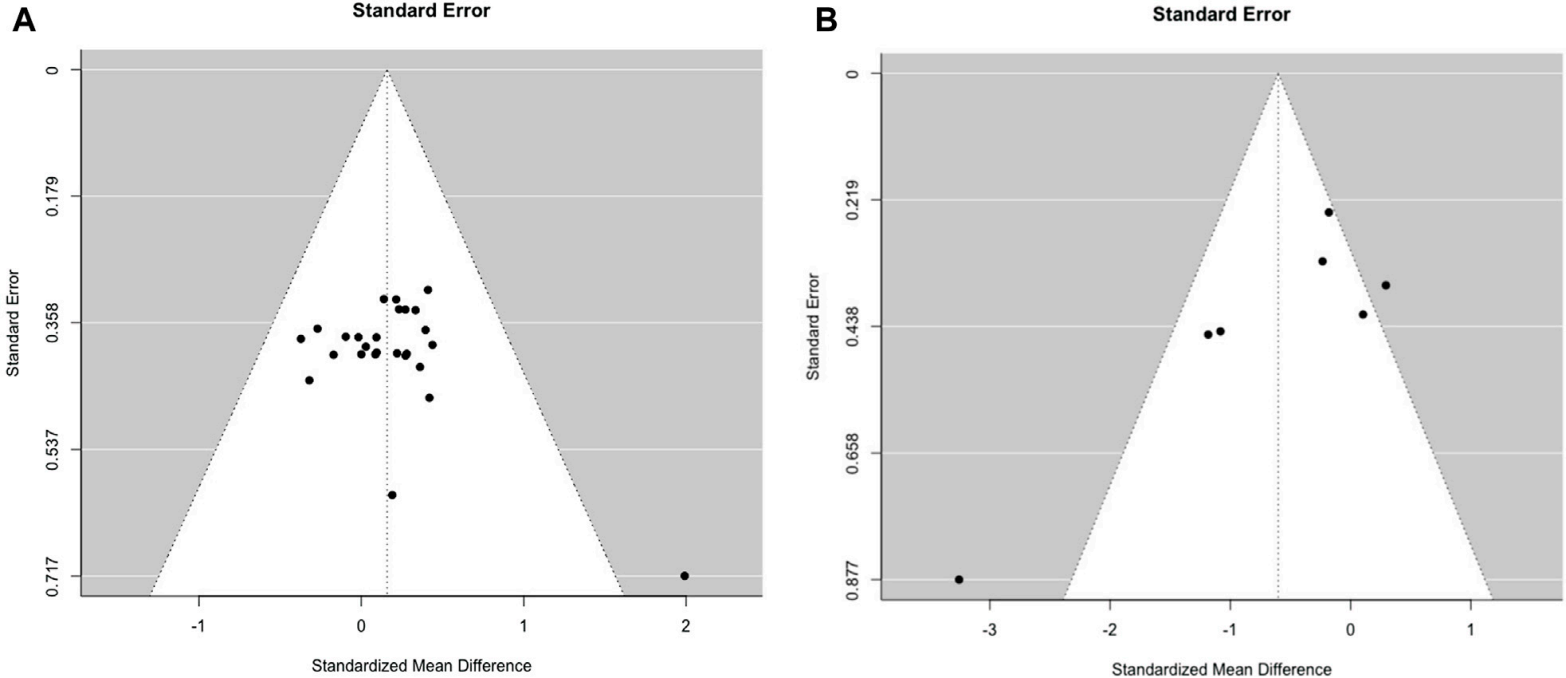
Funnel plots for visual inspection of a potential publication bias for **(A)** chronic stretching on jumping performance and **(B)** chronic stretching on sprinting performance.

Visual funnel plot inspection suggests a publication bias for sprinting performance, while in the jumping performance no asymmetry was observable (Figure 3). With *p* = 0.25 for jumping and *p* = 0.002 for sprinting, the Eggers test confirms these results.

## 4 Discussion

Since recent reviews pointed out chronic stretching programs were sufficient to induce maximal strength increases (Arntz et al., 2023) or muscle hypertrophy (Panidi et al., 2023), this review was conducted to investigate potential positive influence on jump and sprint performance. Fourteen out of 20 studies did not support previously suggested stretch-induced performance enhancements (Shrier, 2004; Medeiros and Lima, 2017), which was confirmed by an overall trivial ES of 0.16 (*p* = 0.04). Similarly, stretching showed no significant benefit for sprinting performance (*p* = 0.078), even though 6 out of 9 included studies indicated a positive influence.

From a physiological point of view, all jumps and sprinting need to apply high forces within a short time period requiring maximal activation via maximal fiber recruitment, frequency, and synchronization (Fleck and Kraemer, 2004; Zatsiorsky et al., 2020). Nevertheless, to account for neuromuscular differences and specific movement execution attributable to, among others, differences in the SSC, subgroup analyses were performed to reduce methodological heterogeneity and to increase comparability of the results. Nevertheless, due to a very limited number of studies to pool effects, training methods heterogeneity remains a limitation of the results. For instance, Donti et al. (2021) and Panidi et al. (2021) included adolescent females, while most other studies explored stretching effects in adults, using untrained (Kokkonen et al., 2007) to highly trained participants (Hadjicharalambous, 2016; Rodriguez Fernandez et al., 2016). Assuming every training program to be effective in untrained populations, the consistent positive direction of the effects for all included tests included by Kokkonen et al. (2007) seems not surprising. Nevertheless, even though some studies showed significant strength increases (Kokkonen et al., 2007; Warneke et al., 2022d), overall, static stretching seems not to induce a sufficient stimulus to enhance jumping and sprinting performance.

### 4.1 The role of maximum strength

According to Keiner et al. (2022a) “strength plays a key role in jumping and sprinting performance, as athletes need to exert great forces within short time intervals during push-off and landing.” Since recent literature showed chronic stretching routines to be sufficient to enhance maximal strength (Arntz et al., 2023) comparable with resistance training programs (Warneke et al., 2023a; Warneke et al., 2023b), a positive influence of athletic performance was reasonably hypothesized. In some of the included studies, maximum strength increases were detected as well. Kokkonen et al. (2007) showed knee extensor and knee flexor strength increases of 32.4% and 15.3%, respectively, while Warneke et al. (2022d) reported plantar flexors strength enhancements of about 10%. Even though strength increases in individual muscle groups have been previously observed, several factors seem to remain unaffected by applying mechanical tension to the muscle via stretching.

### 4.2 Training specificity

Jump and sprint are coordinated power activities involving both strength (force) and velocity. According to the concept of training specificity (Behm and Sale, 1993), training parameters should mimic task or activity characteristics (e.g., velocity, contraction types, movement patterns, joint angles). Whereas static stretching involves elongating soft tissues into a fixed length that is maintained for a prolonged period (i.e., 10–30 s), jumping and sprinting are high velocity stretch-shortening cycle(s) activities of relatively short durations (<1 s per jump or stride) with transition or amortization periods typically less than 250 m (Komi, 1984; Aura and Komi, 1986). Furthermore, jumping and sprinting performance must be considered a much more complex and multi-factorial construct. Assuming a significant correlation and a causal relationship of plantar flexors maximum strength and jumping performance of r = 0.52–0.54 (Warneke et al., 2022b), this correlation accounts for *r* ^2^ = 25–27% of the variance. Whereas static stretching is primarily applied to individual muscle groups in a fixed position (Donti et al., 2021; Nakamura et al., 2021; Panidi et al., 2021; Warneke et al., 2022d), athletic movements consist of a well-coordinated interaction of several muscle groups including specific neuromuscular requirements. Hence, static stretching *versus* jumping and sprinting would elicit very different neuromuscular responses. Jumping and sprinting for maximal performance would involve high motor unit recruitment especially of the type II fast twitch motor units, which would be fired at high firing frequencies, with some degree of synchronization, while coordinating the activation of agonists, antagonists, synergists and stabilizer muscles (Behm, 1995). Although, Budini and Christova. (2023) proposed motor evoked potentials to be greater while stretching, which might attributable to muscle spindle induced corticomotor facilitation, Magnusson et al. (1996) reported no changes in hamstrings EMG activity during a 3 week stretch training protocol. Even with stretch-related increases in sympathetic nerve activity (Cui et al., 2006; Inami et al., 2014), these stretch-induced adaptations seem to be insufficient to affect jump and sprint performance. Hence, since static stretching does not mimic the specific active joint movements of jumping and sprinting, nor evoke maximal muscle activation, trivial to no significant stretching effects on speed strength performance might be attributable to insufficient neuromuscular activation patterns.

### 4.3 Comparison to resistance training routines

Accordingly, performing active multi-joint strength training exercises seems to activate the muscles in a more appropriate way. Referring to high load squat training to beneficially impact sprinting (Lohmann et al., 2022) and jumping performance (Keiner et al., 2022a; Keiner et al., 2022b), using high training intensities in resistance training might require maximal motor unit discharge frequency and synchronization, which is required to explosively accelerate the body (jumping, sprinting). Unsurprisingly, the squat and deadlift showed correlations up to r = 0.91 on vertical jumping height and r = −0.88 on sprinting performance, explaining about 80% of variance (Wisløff et al., 2004; Warneke et al., 2022c). When compared to the effects of resistance training on jumping and sprinting, stretch training effects fared poorly in comparison with trivial and non-significant effects respectively. Accordingly, current meta-analyses demonstrate strength training and plyometrics to be of superior effectiveness with ES = 0.6–0.8 for power training and 0.88–1.35 for strength training (Behm et al., 2017).

In specific training facilities and circumstances, stretching might have some practical applications as an alternative to resistance training (Behm et al., 2023). However, when aiming to improve athletic performance a more potent stimulus seems necessary, which was not apparent with the stretch training investigated in the currently available literature. Assuming a dose-response relationship for stretching volume to enhance maximal strength, it is possible that higher stretching volumes, frequencies (Arntz et al., 2023) or intensities (Panidi et al., 2021) would induce significant adaptations. Accordingly, the comparatively small effect sizes could also explain the lack of significance in the subgroup analyses for jumping performance. While the overall result reached statistical significance, the trivial magnitude effects might be influenced by the inclusion of further studies building a larger participant database.

### 4.4 The potential role of flexibility in speed strength performance

Stretching is well-known for increasing flexibility/range of motion in humans (Young et al., 2013; Medeiros and Martini, 2018). Apart from decreased muscle and/or tendon stiffness (Morse et al., 2008; Takeuchi et al., 2023) and changes in the pain/stretch threshold (Freitas and Mil-Homens, 2015; Freitas et al., 2018), animal stretch training studies demonstrate a significant increase in the number of sarcomeres in series, leading to a greater muscle length (Zöllner et al., 2012; Warneke et al., 2022a). On the one hand, Wirth (Wirth, 2011) described a possible positive influence of sarcomere in series accumulation on speed strength performance (i.e., jumping and sprinting performance) due to an optimized contraction property (Herring et al., 1984) induced by mechanical tension. Accordingly, Kruse et al. (2021) pointed out that enhancements in muscle lengths could improve the muscular energy production capacity with potential implications for power production This suggests that long-term adaptations of stretching might be used to advantage in training routines to increase muscle performance.

On the other hand, negative effects could be expected as recent literature pointed out a reduction in muscle-tendon stiffness (Takeuchi et al., 2023). One important mechanism leading to increased power in the SSC is the elastic kinetic energy storage in the parallel-elastic structures of the muscle tissue and series-elastic components of the tendons, pre-activation of the muscle and reflex properties of the muscle stretch reflex (Komi, 2003). Therefore, Kalkhoven & Watsford (2018) suggest a positive influence of higher stiffness in sub-elite footballers on sport specific performance, including maximal sprinting speed and (drop-) jump performance. Furthermore, Fukashiro et al. (2002) described higher stiffness in black (African ancestry) athletes compared to white (European ancestry) athletes and discussed the contribution of muscle stiffness on ground-contact time and the performance output in SSC determined performance parameters. Assuming reduced muscle stiffness in response to long-term stretching programs (Nakamura et al., 2021; Takeuchi et al., 2023) a negative effect on sport-specific performance including the SSC could be hypothesized. However, Kallerud et al. (2013) could not find any negative influences of long-term static stretching on SSC in their systematic review.

## 5 Limitations

Meta-analytical approaches in sport and exercise science can be controversial since invalid comparisons are sometimes performed, which can skew the conclusions. Nevertheless, pooling study effects increases the sample size and therefore enhances the statistical power. To provide a well-balanced perspective, an extended qualitative description of the study results was provided to account for heterogeneity in methods. The meta-analytical approaches might be seen as a supplementation to quantify results. Although some short duration stretching interventions sufficiently increased maximal strength (Nelson et al., 2012; Mizuno 2019), consistent stretch-induced strength and muscle size increases were reported after longer stretching durations (≥10 min per session) (Chen et al., 2011; Wohlann et al., 2023; Wohlann et al., 2024, Warneke et al., 2022a, Reiner et al., 2023; Warneke et al., 2022). However, stretching durations of the included studies primarily used short stretching durations of a few minutes. Consequently, especially when considering maximal strength was only one component in the multifactorial construct of jumping and sprinting performance, the included stretching studies did not involve stretching volumes that allow a reasonable assumption of increased athletic performance. However, since there were increases in the Warneke et al. (2022b) study using a weekly volume of 70 min of stretching, future research on stretch-induced jumping and sprinting performance enhancements should include larger stretching dosages. Furthermore, pooled linear sprint performance effects originate from different sprinting distances, starting with 4.5 m (acceleration phase) until 55 m (maximum velocity phase). Therefore, different physiological and biomechanical requirements are addressed, thus, the calculated effect size might be considered with care. However, it seems of limited value to divide these studies in further subgroups, as the number of pooled effects was small. In general, the current stretching study situation calls for further research.

## 6 Practical applications and conclusion

To date, no beneficial effects of chronic stretching on jumping and sprinting performance could be detected. Due to the limited number of studies and extracted effect sizes with questionable methodological homogeneity, further research with extended intervention periods and longer stretching durations with high intensities seems necessary, since those provided the highest magnitude maximum strength increases.

## Data Availability

The original contributions presented in the study are included in the article/supplementary material, further inquiries can be directed to the corresponding author.
